# Megadose Methylprednisolone (MDMP) Treatment in a Patient with Autoimmune Hemolytic Anemia (AIHA) Resistant to Conventional Corticosteroid Administration: A Case Report

**DOI:** 10.4274/Tjh.2012.0070

**Published:** 2013-06-05

**Authors:** Şinasi Özsoylu, Henriette WA Berenschot

**Affiliations:** 1 Fatih University, School of Medicine, Department of Hematology, Ankara, Turkey; 2 Albert Schweitzer Hospital, Division of Hematology, Dordrecht, Netherlands

**Keywords:** Autoimmun hemolytic anemia, Megadose methylprednisolone

## Abstract

A female in the Netherlands with severe autoimmune hemolytic anemia (AIHA) was treated with conventional corticosteroid (2 mg/kg/d in divided doses) and blood transfusions for 18 months without improvement. The presented patient responded to megadose methylprednisolone (MDMP) 30 mg/kg/d for 3 d, followed by 20 mg/kg for 4 d, and subsequently 10, 5, 2, and 1 mg/kg/d each for 1 week.

**Conflict of interest:**None declared.

## Abstract (tr)

Ağır otoimmün hemolitik anemili bir kadın hasta 18 ay konvansiyonel kortizon (2 mg/kg/gün bölünmüş dozlarda) uygulamasına cevap vermeyince splenektomi planlandığı için bize başvurdu. Oral megadoz metilprednizolon (MDMP; 30 mg/kg/gün 3 gün, 20 mg/kg/gün 4 gün verildikten sonra haftada 10, 5, 2 ve 1 mg/kg/gün dozlarda) sabah 6’da verilmesi ile kısa sürede Coombs pozitifliği devam etmesine rağmen hemoliz durdu ve hematolojik bulgular düzeldi. Son 18 aydan beri tedavi almayan hastada Coombs pozitifliği devam etmekte ise de hemoliz olmaksızın şikâyetsiz olarak yılda bir kere kontrole gelmek üzere takip edilmektedir.

## INTRODUCTION

A 36-year-old female suddenly developed pallor, tachycardia, fatigue, jaundice, and dark urine. She was diagnosed with as autoimmune hemolytic anemia (AIHA) based on a high reticulocyte count, positive direct Coombs test, hemoglobinuria, and low haptoglobin level, and received the conventional treatment of prednisone (2 mg/kg in divided doses) and blood transfusions. Tests for paroxysmal nocturnal hemoglobinuria were negative, and acquired thrombotic thrombocytopenic purpura and Evans syndrome were excluded. As her low hemoglobin level could not be corrected with prednisone treatment, the symptoms and signs persisted, and transfusion requirement did not decrease during 18 months, the possibility of splenectomy was discussed with her. She was then referred to us for meagdose methylprednisolone (MDMP) treatment before splenectomy [1,2,3,4,5]. 

The patient presented to us for the first time on 18 March 2006 in Ankara after having received transfusion. She appeared pale, depressed, cushingoid, and jaundiced, which was more severe in the sclerae. Her blood pressure was 106/65 mm/Hg, pulse rate was 112/min, and temperature was 36.5 °C. Her spleen and liver were 2 cm and 1 cm below the respected costal margins. Her Hb level was 8.2 g/dL, Hct was 26%, and reticulocyte count was >20%. Peripheral blood smear showed several spherocytes and macrocytes, and polychromasia and anisocytosis were noted. Hemoglobinuria (not hematuria), unconjugated bilirubinemia (3.35 mg%), elevated LDH (491 U/L), normal vitamin B12 (439 pg/mL), and normal folic acid (13.24 ng/mL) were noted; therefore, her macrocystosis (MCV: 106.7 fL) was considered to be associated with her marked reticulocytosis.

She was started on our MDMP treatment regimen, as follows: 30 mg/kg/d for 3 d, followed by 20 mg/kg/d for 4 d, and then 10, 5, 2, and 1 mg/kg/d each for 1 week [1]. Each dose was given orally at about 0600 by placing the methylprednisolone (MP) powder in a tablespoon and covering it with honey, as the taste of MP is extremely bitter. Dark urine due to hemoglobinuria disappeared on d 3 of treatment and scleral jaundice cleared markedly after 1 week, along with a decrease in bilirubinemia (1.86 mg/dL). Her hemoglobin started to increase (Hb: 9.2 g/dL; Hct: 28.4%) during the first week of MDMP treatment as her reticulocyte count decreased (8.19%). Her early morning blood sugar was high (152 mg/dL), most likely due to ingestion of honey. She was administered saline nose drops to prevent of upper respiratory tract infection [7,8] and she was directed to walk rapidly as much as possible to prevent muscle atrophy, in addition to performing bicycling-like leg exercises when lying down.

Her Hb (12.8 g/dL) and Hct (39%) returned to normal during the second week of the treatment, along with normalization of the haptoglobin level (1.4 g/L) and a decrease in the reticulocyte count (2.7%). Her spleen was non-palpable and urine glucose was positive (4+) without ketonuria; however, blood electrolytes, lipids, and pH levels were within normal limits; therefore, they were related to honey ingestion during the very early MDMP administration.

The patient began insulin (20 IU) and oral antidiabetic (glutazione t.i.d.) treatment when she was in the Netherlands prior to presentation. As her fasting blood glucose was 125 mg/dL before honey ingestion with MP, and her blood electrolytes, lipids, and pH were normal, insulin was discontinued the 8th week of the treatment. She started to complain about proctatatis, and a perianal abscess was diagnosed and drained the 10th week of the treatment, during which time she was taking MP 64 mg/d in the early morning. In addition, her oral antidiabetic treatment was discontinued at the same time, as she had only minor symptoms of hypoglycemia. She again appeared mildly cushingoid the 11th week of treatment, at which time her Hb (13.3g dL), Hct (39%), and MCV (92.1 fL) were normal, and her reticulocyte count (3.12%) was slightly elevated, and she was taking only MP 48 mg/d; her early blood sugar was 141 mg, but HbA1c was normal (3.97%) at that time. Her peripheral blood smear was negative for spherocytes and polychromasia, despite Coombs positivity.

On the 6th month of treatment despite normal Hb (14.6 g/dL), Hct (42.1%), MCV (90.1), and reticulocyte count (1.34%) steroid-induced glaucoma was suspected. The MP dose was decreased (16 to 32 mg every other d) and eye drops (Betoptic) were prescribed. The MP dose was tapered to 16 mg/d on the 8th month of the treatment. During the 14th month of MDMP treatment she was taking MP 16 mg every 5 d, despite questionable direct Coombs positivity. She has been engaged in normal daily activity without dietary restriction, and her blood pressure was 98/62 mm/Hg. She does not have eye complaints and her eye tension has been almost normal during the last 4 months. At the time this report was prepared the patient was still using serum physiologic nose drops t.i.d. Her MP treatment was discontinued after 61 months of treatment despite Coombs positivity; however, her Hb level remained normal without any sign of hemolysis during the last 27-month period and she has had normal blood glucose for more than 5.5 years.

## DISCUSSION

The presented patient is a good example that illustrates the necessity of giving corticosteroids once a day in the early morning, so as not to disturb ACTH corticosteroid homeostasis and prevent their side effects. With the exception of adrenal insufficiency divided corticosteroid doses should not be given. It is also important to note that MDMP treatment differs from pulse MP treatment (in which MP 1000 mg is given intravenously at any time of the day within 4 h) and conventional corticosteroid treatment, both of which can suppress ACTH secretion, the main cause of steroidal side effects [6].

Steroid-induced glaucoma was suspected in the presented patient in about the 7th month of MDMP treatment, but her eye pressure was not evaluated prior to MDMP treatment though she had some vision complaints at presentation. As such, we repeatedly monitored for cataract, but the complaints were related to hypotension. Most importantly, her steroid induced diabetes was cured with MDMP, making her the second patient in which corticosteroid induced diabetes was cured during MDMP treatment [9]. Neither electrolytes nor lipid fraction disturbances were observed in the presented patient during hyperglycemic period, which was controlled without dietary or salt restriction as the MDMP dose decreased, as in our previous patient. Additionally, her chronic AIHA came under control after 1 week of MDMP treatment, hemoglobinuria resolved and the bilirubin level decreased after 3 d of MDMP; the only side effect was a mild cushingoid appearance that regressed after 2 months of the treatment, despite continuation of a relatively low dose of MP (48 mg/d).

MDMP treatment has been used to successfully treat more than 700 patients with different hematological and non-hematological disorders in whom corticosteroid administration was advised, and we have shown that MDMP treatment does not impair the growth of patients, even with long-term administarion [2,10]. Others [11] also supported our observations that corticosteroid side effects practically are not seen with our way of MDMP administration, such as hypertension, obesity and suppression of growth in children. MDMP has been used to treat AIHA patients [3,5]. Coombs test results were reported to have become negative in more than 4 cases with improvement of anemia in all patients [3,12] with prolonged administration of MDMP treatment, as in the presented patient. Also of importance is patient activity during MDMP treatment for the prevention of muscle atrophy and the use of saline nose drops for the prevention of upper respiratory system infections. We think that in addition to a decrease in antibody formation, an increase in the erythropoietin level with MDMP should be considered in the improvement of anemia Table 1.

## CONFLICT OF INTEREST STATEMENT

The authors of this paper have no conflicts of interest, including specific financial interests, relationships, and/ or affiliations relevant to the subject matter or materials included.

## Figures and Tables

**Table 1:**
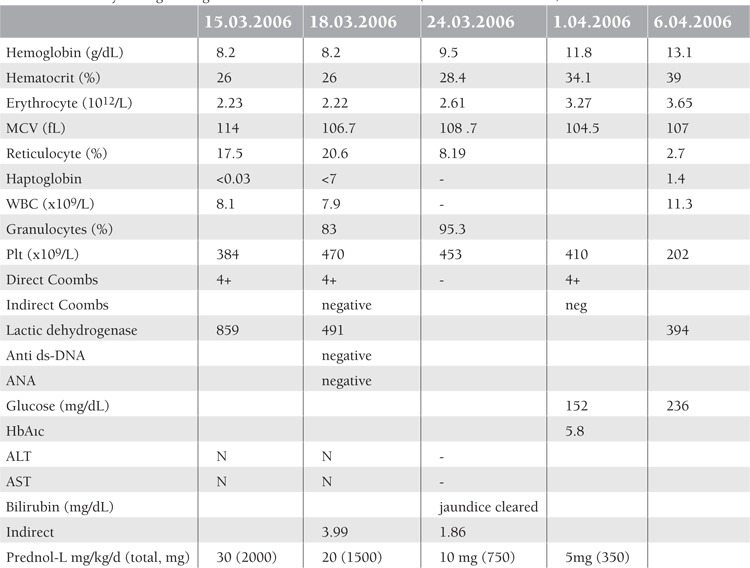
Laboratory findings during the first month of MDMP treatment (doses are also indicated).
